# Differential macrophage function in Brown Swiss and Holstein Friesian cattle

**DOI:** 10.1016/j.vetimm.2016.02.018

**Published:** 2016-11-15

**Authors:** Amanda Jane Gibson, Sally Woodman, Christopher Pennelegion, Robert Patterson, Emma Stuart, Naomi Hosker, Peter Siviter, Chloe Douglas, Jessica Whitehouse, Will Wilkinson, Sherri-Anne Pegg, Bernardo Villarreal-Ramos, Dirk Werling

**Affiliations:** a Molecular Immunology Group, Department of Pathology and Pathogen Biology, Royal Veterinary College, Hawkshead Lane, North Mymms, AL9 7TA, UK; b Animal and Plant Health Agency, Bovine TB, Weybridge, New Haw, Addlestone, Surrey KT15 3NB, UK

**Keywords:** Innate immunity, Cattle breeds, ROS, Inflammasome, Macrophages

## Abstract

There is strong evidence that high yielding dairy cows are extremely susceptible to infectious diseases, and that this has severe economic consequences for the dairy industry and welfare implications. Here we present preliminary functional evidence showing that the innate immune response differs between cow breeds. The ability of macrophages (MØ) to kill pathogens depends in part on oxygen-dependent and independent mechanisms. The oxygen-dependent mechanisms rely on the generation of reactive oxygen and nitrogen species (ROS/RNS, respectively). ROS production has been shown to activate the inflammasome complex in MØ leading to increased production of the pro-inflammatory cytokine Interleukin-1β (IL-1β). Conversely RNS inhibits inflammasome mediated IL-1β activation, indicating a division between inflammasome activation and RNS production. In the present study MØ from Brown Swiss (BS) cattle produce significantly more RNS and less IL-1β when compared to cells from Holstein Friesian (HF) cattle in response to bacterial or fungal stimuli. Furthermore, BS MØ killed ingested *Salmonella typhimurium* more efficiently, supporting anecdotal evidence of increased disease resistance of the breed. Inhibition of autophagy by 3-methyladenine (3-MA) stimulated IL-1β secretion in cells from both breeds, but was more pronounced in HF MØ. Blocking RNS production by l-arginase completely abolished RNS production but increased IL-1β secretion in BS MØ. Collectively these preliminary data suggest that the dichotomy of inflammasome activation and RNS production exists in cattle and differs between these two breeds. As pattern recognition receptors and signaling pathways are involved in the assessed functional differences presented herein, our data potentially aid the identification of *in vitro* predictors of appropriate innate immune response. Finally, these predictors may assist in the discovery of candidate genes conferring increased disease resistance for future use in combination with known production traits.

## 1 Introduction

Macrophages (MØ) are the mature form of circulating monocytes (Mo) derived from a common myeloid progenitor. Inflammatory cytokines trigger adherence and rolling of circulating Mo along the endothelium of adjacent blood vessels before extravasation, maturation to MØ and migration to sites of inflammation, where they have both pro-inflammatory and inflammation resolution activity (Aguilar-Ruiz et al., 2011, Cros et al., 2010, Hussen et al., 2013, Shi and Pamer, 2011, Wong et al., 2011, Wong et al., 2012). In humans Mo can be typified into three subsets based on the expression of CD14 and CD16: CD14++CD16−, CD14++CD16+ and CD14+CD16++. These are referred to as classical (cM), intermediate (intM) and non-classical (ncM), respectively (Ziegler-Heitbrock et al., 2010). It has been indicated that the three subsets have different roles (Grage-Griebenow et al., 2001) and there may be a developmental pathway from cM through intM to ncM (Wong et al., 2011, Zawada et al., 2011). Exposure to the cytokine Macrophage-Colony Stimulating Factor (M-CSF) may trigger this development (Korkosz et al., 2012).

In cattle, approximately 89% of monocytes are cM (Hussen et al., 2013). These express greater phagocytic activity than intM or ncM, whilst also producing anti-inflammatory cytokines such as IL-10 (Grage-Griebenow et al., 2001, Ziegler-Heitbrock, 2007, Ziegler-Heitbrock et al., 2010) and being involved in repair of damage tissue (Wong et al., 2011, Wong et al., 2012). Both subsets of CD16+ Mo increase with infection and disease, extravasating more efficiently and producing cytokines including TNFα and IL-1β, thus being described as inflammatory Mo (Aguilar-Ruiz et al., 2011, Ancuta et al., 2003, Belge et al., 2002, Grage-Griebenow et al., 2001, Tacke and Randolph, 2006, Thieblemont et al., 1995, Wong et al., 2012, Zawada et al., 2011, Ziegler-Heitbrock et al., 2010), although some findings indicate anti-inflammatory actions for ncM (Cros et al., 2010, Hussen et al., 2013). A range of pro-inflammatory actions are exhibited by murine and human intM, from inflammatory cytokine production to antigen presentation, requiring prior phagocytosis (Aguilar-Ruiz et al., 2011, Belge et al., 2002, Shi and Pamer, 2011, Thomas and Lipsky, 1994, Ziegler-Heitbrock, 2007).

Correlating with their role in antigen presentation, human intM display the highest MHC II expression levels of the three monocyte subsets (Wong et al., 2011), indicating a strong involvement in antigen presentation. In addition to the production of cytokines and their function as antigen presenting cells, MØ constantly sample the local environment and have antigen presenting capacity (Chesnut and Grey, 1985, Gordon, 1998, Gordon and Taylor, 2005).

Responses to pathogens by MØ are mediated by pattern recognition receptors (PRR) such as Toll-like Receptors (TLR) which recognise and interact with pathogen associated molecular patterns (PAMP) (Akira et al., 2001, Taylor and Gordon, 2003). PAMPs include, but are not restricted to pathogen surface structures, genetic material and secreted or released products (Taylor and Gordon, 2003). MØ are characteristically bactericidal in nature and utilise several approaches to mediate bacterial killing but mainly employing two mechanisms which are closely interlinked: production of reactive oxygen species and reactive nitrogen species (ROS and RNS, respectively). Their release results in cell- and DNA damage and thus shows antimicrobial effects (Bogdan, 2001, Forman and Torres, 2001, Nathan, 1992, Nathan, 1987). Interaction of a PAMP with the corresponding PRR stimulates a variety of pathways, resulting in the activation with the subsequent cleavage of pro-IL-1β into its active, secreted form by caspase-1 (Lamkanfi et al., 2007), assembly of the NADPH oxidase complex, electron transfer from NADPH to oxygen and a series of enzymatic reactions to produce ROS (Bokoch and Knaus, 2003, Lambeth, 2004, Quinn and Gauss, 2004). Additionally, PAMP association with PRR results in transcription of inducible Nitric Oxide Synthase (iNOS) in MØ which interacts with NADPH and ROS intermediates to create RNS in bovine and murine MØ (Jungi et al., 1996a, Nathan, 1992, Green et al., 1990).

However, there are some infectious diseases where – due to the anatomical barrier present – the anti-bacterial activity of MØ is not sufficient to eliminate the pathogen. One such organ is the bovine udder, where mechanical, chemical and pathological irritation can lead to an increased somatic cell count (SCC) (Schukken et al., 2003). However, it has to be emphasised that somatic cells in the milk consist of a pleiotropha of cells, including MØ, neutrophils, lymphocytes and epithelial cells (Boulanger et al., 2001). In humans, the majority of cells in milk have been identified as unique breast milk MØ (Yagi et al., 2010). These have been shown to be more metabolically active compared with those in peripheral blood (Johnson et al., 1980). However, the killing mechanism was shown to involve the oxidation of glucose via the hexose-monophosphate (HMP) shunt, showing that cytotoxic effects are using the same mechanism as blood derived cells by the production of hydrogen peroxide and superoxide (Johnson et al., 1980). Indeed, bovine mammary MØ challenged with phagocytic stimuli produced ROS similarly to those produced by other MØ (Harmon and Adams, 1987). Thus milk-derived MØ are capable of ROS/RNS production, has been described in milk-derived MØ (Denis et al., 2006), and seems to be an approach of the innate immune cells to combat invading bacteria in the udder.

However, despite the fact that 1/4 of cows are suffering from mastitis at any given time, not all breeds show the same mastitis incidence rate (Begley et al., 2009). Indeed, *c*attle breed susceptibility or resistance to infection has long been studied to improve breeding strategies (Warner et al., 1987), and differences in the genetic resistance to infection have been identified for *Mycobacterium tuberculosis* (O’Reilly and Daborn, 1995, Vordermeier et al., 2012) and *Brucellosis* (Paixao et al., 2006).

However, it has to be emphasised that research results, especially analysis of immune responses, may not translate between breeds of the same species. Indeed, observations by farmers and veterinarians have noted that Brown Swiss (BS) cattle tend to have a lower somatic cell counts (SCC) than Holstein-Friesian (HF) cattle, and are thought to be less susceptible to mastitis. This anecdotal evidence has been reinforced by studies suggesting differences in SCC could be the result of improved microbial killing mechanisms in the innate cells of BS cattle (Busato et al., 2000, Kizilkaya, 2009, Rupp and Boichard, 2003). Work by Norimatsu et al. (2004), showing low RNS production from *Salmonella typhimurium* stimulated HF MØ compared to data published by Werling et al. (2004) demonstrating high RNS production from BS MØ with the same bacterium highlights that difference in disease resistance may be conferred by the micro-bicidal capabilities of diverse breeds. Furthermore, work by Jann et al., 2008, Jann et al., 2009 suggests that polymorphisms in TLR genes may be involved in disease resistance or susceptibility traits in domestic animals (Jann et al., 2009, Jann et al., 2008). Despite estimations in heritability of production traits varying (Rupp and Boichard, 2003), Lund et al. (1994) have shown the estimated heritability of SCC to be moderately high (Lund et al., 1994). This suggests the possibility of integrating low SCC into breeding strategies, particularly if lower SCCs in milk could be linked to an increased MØ killing ability.

## 2 Materials and methods

### 2.1 Sample collection

Blood for PBMC isolation and subsequent MØ generation was collected by puncture of the jugular vein from clinically healthy HF and BS cows housed at either the RVC Boltons Park Farm (Hertfordshire, UK) or at Cancourt Farm (Wiltshire, UK). Animals used were age- and lactation-matched (2nd or 3rd lactation, respectively), unless otherwise stated. All procedures were carried under Home Office Project licence which was approved by the College’s Ethics and Welfare Committee. For biological assays, blood was drawn into sterile glass vacuum bottles containing 10% Acid Citrate Dextrose (ACD) as anticoagulant. For whole blood flow cytometric analysis, blood was collected from three cows of each breed into 10 ml EDTA Vacutainers (Beckton Dickinson, Oxford, UK) by puncture of the jugular vein. Whole milk was collected into sterile glass bottles from individual cows housed at either farm. All assays were performed in at least four breed matched pairs apart from bacterial killing assays, blood MØ NO induction and preliminary IL-1β stimulations, milk derived responses which were performed in 3, 2 and 1 breed matched pairs, respectively.

### 2.2 Isolation peripheral blood mononuclear cells and generation of macrophages

Peripheral blood mononuclear cells (PBMC) were isolated from whole blood as previously described (Jungi et al., 1996b). Briefly, blood was centrifuged at 700* × g* for 20 min before buffy coats were removed and washed in citrate buffer. RBC lysis was performed using ammonium-chloride lysis buffer. Resultant cells were suspended in RPMI media (Life Technologies, UK) before being underlayed with Histopaque (*d* = 1.083 g l^−1^, Sigma–Aldrich, UK) to isolate PBMCs by density centrifugation. PBMC were washed in PBS and counted by Trypan blue exclusion then incubated in Teflon bags for 7 days at 37 °C and 5% CO_2_ at a concentration of 5 × 10^6^ ml^−1^ in MØ medium containing 10% FCS. To harvest MØ, Teflon bags were placed in the fridge at 4 °C for 30 min and agitated to loosen cells. Cells were washed three times in PBS at 300 × *g* for 10 min, before counting as described above.

### 2.3 Determination of circulating monocyte populations

Mo populations were determined as previously described with modifications (Hubl et al., 2000). Briefly Briefly 50 μl blood samples were labelled with 5 μl mouse anti-human (mαh) CD14 APC (AbD Serotec, UK), 5 μl mαh CD16 FITC (AbD Serotec, UK) and 15 μl mαb BoLA-DR (clone CC108) labelled with PE-680 via Zenon labelling as per manufacturer’s instructions. Isotype controls were prepared by direct addition of 10 μl Mouse IgG_2a_ APC, 10 μL Mouse IgG_2a_ FITC and 20 μl Mouse IgG_1_ labelled with PE-680 as above to 50 μl of whole blood. Antibodies were allowed to incubate at room temperature for 20 min in the dark. Red blood cells were lysed by addition of ammonium chloride lysis buffer for 10 min as above before placing on ice. Remaining cells were washed in PBS (Gibco, UK) and suspended in FACSFlow (BD Biosciences, UK) for analysis. Samples were analysed by FACS Calibur (BD Biosciences, UK). Mo populations were gated for the presence of BoLA-DR and CD14 to exclude platelets, lymphocytes and granulocytes. BoLA-DR^hi^ or CD14+ populations were used to identify CD14+ and CD16+ population proportions of cM, intM and ncM. 50,000 events were recorded per sample using Cell Quest (BD Biosciences, UK). Data were subsequently analysed using FCS Express 4 (DeNovo Software, USA).

### 2.4 Isolation of bovine macrophages from milk

Whole milk samples were kept on ice for 10 min prior to centrifugation at 3000 × *g* for 5 min at 4 °C. Residual fat layer was removed using a spatula and supernatant discarded. Cell pellets were re-suspended in PBS/0.125 M EDTA/TE buffer (10 mM—Tris–HCl–1 mM EDTA pH 7.6) and allowed to stand at room temperature for 10 min. Cells were washed twice in TE buffer and finally suspended in PBS (PAA Laboratories, UK) for counting by Trypan blue exclusion as above.

### 2.5 Measurement of ROS production

ROS was measured as described previously (Conejeros et al., 2011). Briefly, production of ROS by MØ was measured by oxidation of 2,7-dichlorofluorescein diacetate (DCFH-DA) to fluorescent 2,7-dichlorofluorescein (DCFH). MØ were harvested as above and adjusted to a concentration of 1 × 10^7^ per condition in HBSS (Gibco) before the addition of stimuli (1 mg ml^−1^ zymosan (Sigma); 2–5 μg ml^−1^ PMA (Sigma), 20 μg ml^−1^ *Listeria monocytogenes* (*L.m.*); 1 × 10^8^ bacteria ml^−1^*, Staphylococcus aureus* (*S.a.*) *ST398* or HBSS as a negative control). DCFH-DA probe (1 mg ml^−1^) was added at final concentration of 100 μg ml^−1^ before being incubated in the dark at 37 °C for 15 min. 150 μl aliquots were plated in triplicate on a black 96-well plate (Corning, UK). RFU were measured using a spectrophotometer (SpectraMax M2, Molecular Devices, UK) at 485 nm excitation and 530 nm emission. Cells were incubated at 37 °C, 5% CO₂ between readings in 15 min intervals for a two hour period.

### 2.6 Measurement of NO production using griess reagents

Determination of NO concentration in cell supernatants was carried out using Griess reagents as previously described (Werling et al., 2004). MØ were harvested as above and adjusted to 4 × 10^5^ cells ml^−1^ in 2% FCS MØ medium and 8 × 10^4^ cells added a flat-bottomed 96-well plate for 24 h at 37° C, 5% CO₂. Adherent cells were washed with PBS after 24 h to remove contaminating lymphocytes. Stimulants were prepared in 200 μl of 2% FCS MØ medium to give a final concentration of 250 μg ml^−1^ zymosan (Sigma, UK), 100 μg ml^−1^ *L.m.*, 5 μg ml^−1^ LPS or media controls. MØ were cultured for a further 24 h 37° C, 5% CO₂ and cell free supernatants were harvested and stored at −20° C until required for measurement of NO or cytokine analysis (IL-1β). Briefly, a two-fold standard curve of 128 μM sodium nitrite in 2% FCS MØ media and sample supernatants were placed in flat 96-well clear plate and mixed with equal volumes of Griess reagent (1:1 solutions A and B). After 10 min incubation absorbance was analysed at 550 nm using a spectrophotometer (Spectramax M2, Molecular Devices, UK).

### 2.7 Inhibition of RNS production by l-arginase

MØ were generated and harvested as described above, adjusted to 1 × 10^6^ ml^−1^ in 2% FCS MØ medium and 2 × 10^5^ MØ were added per well of 96-well plate and cultured as above. Adherent cells were washed after 24 h twice with PBS before addition of 2% MØ media, RNS inhibitor l-arginase (0.125 μg ml^−1^) and Zymosan (1 mg ml^−1^). Supernatants were collected 24 h later and stored until analysis of RNS and IL-1β production as described.

### 2.8 Inhibition of autophagy in macrophages with 3-methyladenine

MØ were generated and harvested as described above, adjusted to 1 × 10^6^ ml^−1^ in 2% FCS MØ medium and 2 × 10^5^ MØ were added per well of 96-well plate and cultured as above. After removal of contaminating lymphocytes and washing adherent cells twice in PBS, MØ were exposed to LPS (5 μg ml^−1^) (Sigma, UK) and two-fold dilution of 3-methyladenine (3-MA) (Sigma, UK) from 10 mM to 1.25 nM, including medium, LPS and 3-MA alone controls. MØ were cultured for a further 24 h at 37° C, 5% CO_2_ and cell free supernatants harvested and stored at −20 °C until assayed for IL-1β by ELISA.

### 2.9 IL-1β detection by ELISA

Cell-free supernatants were stored at −20 °C until sandwich ELISAs could be used to analyse cytokine production (IL-1β) in response to the ligand sets described (Zymosan, *L. monocytogenes* and LPS). ELISA kit specific for boIL-1β (Thermo-Scientific, IL, USA) was prepared using a Maxi-sorb 96-well ELISA plate (Nunc, Sigma, UK) and performed according the manufacturer’s instructions. Samples were tested in duplicate and standard curves were generated using a serial 1:2 dilution from 2 ng ml^−1^ IL-1β. Samples diluted 1:10 with reagent diluent were assayed alongside standards as per manufacturers instructions without modifications. After final addition of stop solution, absorbance was measured at 450 nm with 550 nm reference using a spectrophotometer (SpectraMax M2, Molecular Devices, UK).

### 2.10 Macrophage phagocytosis of *S. aureus* bio-particles

MØ were harvested as described above and adjusted to 1 × 10^6^ ml^−1^ in RPMI 1640 medium without phenol red (Gibco, UK) before placing 2 × 10^5^ MØ per well of a 96 well U-bottomed low bind plate (Costar, USA). MØ were exposed to *S.a.* BioParticles^®^ Alexa-594 (Life Technologies, UK) and were incubated at 37 °C and 5% CO_2_. At two different timepoints (0 and 60 min) 100 μl of 4% paraformaldehyde was added to fix the cells and halt phagocytosis. Cells were then washed three times in PBS (Gibco, UK) and suspended in FACS flow for analysis. Samples were analysed by FACS Calibur (BD Biosciences UK), data collected with Cell Quest Pro (BD Biosciences, UK) and subsequently analysed using FlowJo v10 (Treestar, USA).

### 2.11 Preparation of *S. typhimurium* cultures

*S. typhimurium* SL1344 was inoculated onto LB agar and incubated at 37 °C for 16–18 h before storing at 4 °C as a source for future liquid cultures. On the day prior to each MØ infection, a new inoculum was created from a single colony in 10 ml sterile LB broth (Oxoid, UK) and cultured overnight at 37 °C for 16–18 h with agitation at 180 rpm. Cultures were centrifuged for 10 min at 3219 × *g*, 21 °C and suspended in RPMI to generate an OD_600_ reading between 0.8–1.0 (the equivalent of 10^8^–10^9^ CFU). Confirmation of CFU was determined by 10-fold serial dilutions of overnight culture plated onto LB agar, cultured overnight at 37 °C and counted.

### 2.12 Infection of macrophages with *S. typhimurium*

MØ were generated and harvested as described above, adjusted to 1 × 10^5^/1 × 10^6^ MØ ml^−1^ in antibiotic free 2% FCS MØ medium and seeded at 1 × 10^5^/1 × 10^6^ per well of 24-well plate for 24 h 37 °C, 5% CO_2_. Contaminating lymphocytes were removed and cells were washed in PBS prior to inoculation with an MOI of 10 for 1 h at 37 °C, 5% CO_2_. After 1 h incubation RPMI supplemented with 10% FCS and 50 mg ml^−1^ Gentamicin was added to eliminate extracellular bacteria and cells were incubated overnight at 37 °C, and 5% CO_2_. Supernatants were collected after 1 h and MØ were washed with PBS before addition of culture medium for a further 24 h of culture at 37 °C, and 5% CO_2._ Supernatants were harvested and MØ were washed with PBS prior to lysis using PBS +1% Triton X for 5 min. Supernatants from 1 h, 24 h and MØ lysis were serially diluted 10-fold before plating on LB agar for 24 h at 37 °C to determine bacterial uptake and killing.

### 2.13 Statistical analyses

Statistical analyses were performed using GraphPad Prism 6 using one-way ANOVA or two-way ANOVA (repeated measures for ROS assays) with Bonferroni post-*hoc* testing. Unpaired *t*-tests were used for analysis of bacterial killing with Bonferroni post-*hoc* testing. Significance was defined as *p* ≤ 0.05 (*), *p* ≤ 0.01 (**), *p* ≤ 0.001 (***) and *p* ≤ 0.00001 (****).

## 3 Results

### 3.1 SCC, but not milk production or monocyte subsets differ between cells isolated from both breeds

**Fig. 1:**
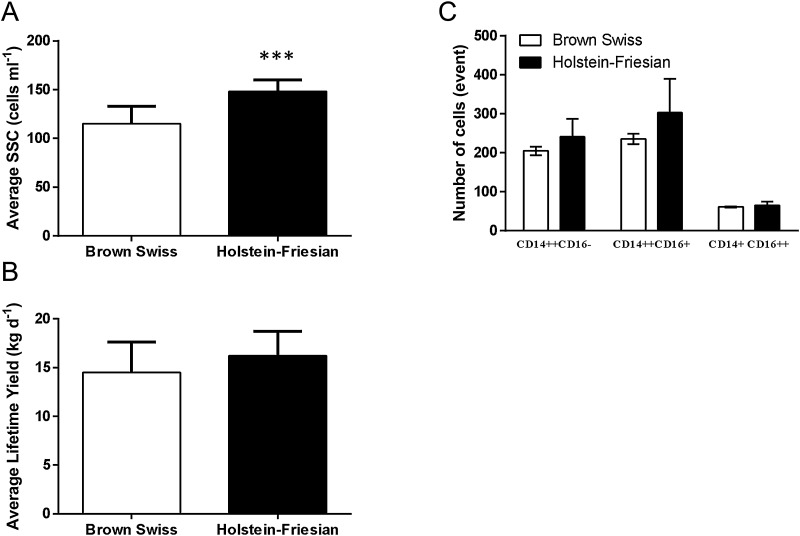
SCC, but not milk production or monocyte subset distribution differ between Brown Swiss and Holstein Friesian cattle. SCC was assessed over a five year period. Data were provided by the National Milk Record, and an annual average of BS and HF animals on the farms is shown (Fig. 1A). In contrast, average milk production (Fig. 1B) as well as composition of Mo subsets (Fig. 1C) was not statistically different. Whole blood from BS and HF cattle were labelled with antibodies directed to human CD14:APC, human CD16:FITC and bovine HLA-DR: PE-680 alongside isotype controls as described. Cells were visualised post RBC lysis and washing using BD FACS Calibur, recording 50,000 events per sample with Cell Quest. Mo subsets were gated from HLA-DR^hi^ and CD14^+^ cells to identify cM (CD14^++^/CD16^−^), intM (CD14^++^/CD16^+^) and ncM (CD14^+^/CD16^++^) population proportions. Data were analysed by FCS Express 4 and population proportions exported to GraphPad Prism for statistical analysis. Differences between breeds were tested by one-way ANOVA, followed by Bonferroni *t*-test (*** *p* < 0.001)

Initially, it was assessed whether the above mentioned “resistance” to mastitis in BS compared to HF was purely based on differences in milk production, SCC or the number/composition of Mo subsets in the peripheral blood. Whereas SCC of BS cows was significantly lower over a one year period (Fig. 1A), neither milk production (Fig. 1B) nor number/composition of Mo subsets (Fig. 1C), as analysed by flow cytometry differed between HF and BS cows.

### 3.2 Ex-vivo blood derived macrophages from Brown Swiss cattle differentially produce ROS and NO compared to Holstein Friesian macrophages

**Fig. 2:**
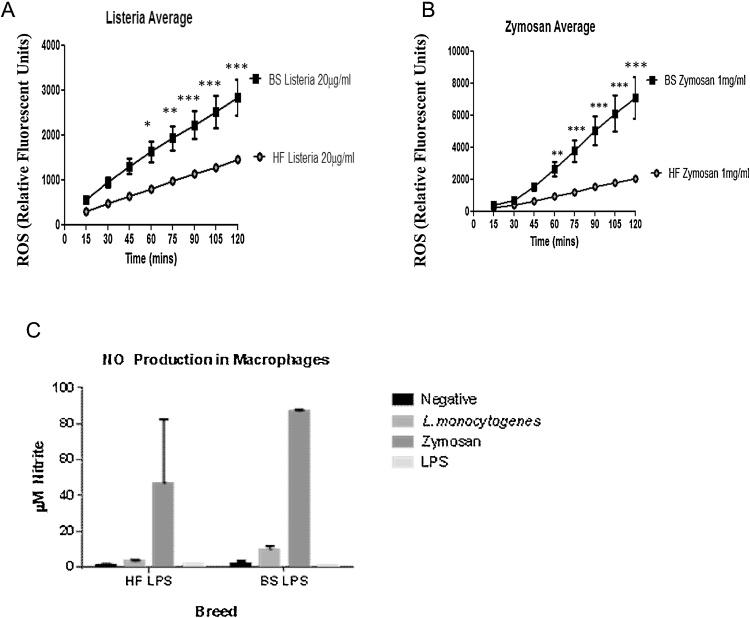
Ex-vivo blood derived macrophages from Brown Swiss cattle differentially produce ROS and RNS compared to Holstein Friesian macrophages. MØ were generated as described from BS and HF PBMC isolated from whole blood. ROS production was measured from MØ exposed to (A) *L. monocytogenes* (1 × 10^8^ bacteria ml^−1^) and (B) zymosan (1 mg ml^−1^), by addition of DCFH-DA at 15 min intervals over 120 min period as relative fluorescent units. Cells were maintained at 37 °C, 5% CO_2_ throughout. Fluorescence was detected using SpectraMax M2 plate reader in black, glass bottomed micro-titre plates. RNS production was measured using Griess assay on overnight macrophage culture supernatant from both cattle breeds. Adhered MØ were exposed to (C) 2% medium, *L. monocytogenes* (100 μg ml^−1^), zymosan (250 μg ml^−1^) and LPS (5 μg ml^−1^) for 24 h before addition of equal volumes of Griess reagent A and B and compared to 2-fold Nitrite standard curve. Absorbance was measured using a SpectraMax M2 plate reader. All data were post analysed with GraphPad Prism using two-way repeated measured ANOVA (ROS) with Bonferroni post-*hoc* correction and two-way non-repeated measures ANOVA (NO) with Bonferronic post-*hoc* testing.

Having established that the potential differences between BS and HF cows are not due to differences in milk production (and thus potentially less cells available) or differences in Mo subsets between both breeds, we next assessed direct anti-bacterial effector functions of matured MØ. Oxidative burst was found to be elevated in BS MØ exposed to *L.m.* and zymosan when compared to HF (Fig. 2A and B). ROS were produced in significantly greater amounts from 60 min post-stimulation for the duration of the assay (to 120 min). Reactivity appears to be stimulation specific and not an overall ‘hyper’ or constitutive activity as MØ from both breeds respond similarly to media or PMA controls (data not shown). Interestingly differences in NO production are less apparent towards the same ligands from the animals tested here, however BS MØ responses show reduced variation (Fig. 2C). Having established differences in ROS/NOS production to the same ligand in MØ derived from both breeds, we next wanted to assess whether these differences also exist in MØ derived from other tissues.

### 3.3 Milk resident cells mirror ex-vivo derived macrophage responses: Brown Swiss cattle produce more ROS Holstein Friesian macrophages

**Fig. 3:**
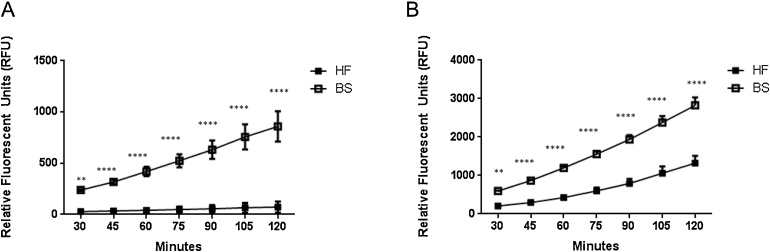
Milk resident cells mirror ex-vivo blood derived macrophage responses: Brown Swiss cattle produce more ROS Holstein Friesian macrophages. MØ from milk were isolated as described from BS and HF cattle, ROS was measured from MØ exposed to (A) *S. aureus* (1 × 10^8^ ml^−1^) and (B) Zymosan (1 mg ml^−1^) by addition addition of DCFH-DA at 15 min intervals over 120 min period. Cells were maintained at 37 °C, 5% CO_2_ throughout. Fluorescence was detected using SpectraMax M2 plate reader in black, glass bottomed micro-titre plates. All data were post analysed with GraphPad Prism using two-way repeated measures ANOVA testing with Bonferroni post-*hoc* correction.

A comparison of ROS production from milk derived cells revealed enhanced responses from cells derived from BS cattle when stimulated with *S.a.* and zymosan as compared to cells derived from, HF cattle (Fig. 3A and B). Increased levels of ROS observed for BS cells is not attributed to increased SCC as over the study period HF milk contained significantly greater somatic cells (Fig. 1A) and equal cell concentrations where stimulated per breed.

### 3.4 RNS blockade partially restores IL-1β Brown Swiss levels to those similar to Holstein Friesian macrophages

**Fig. 4:**
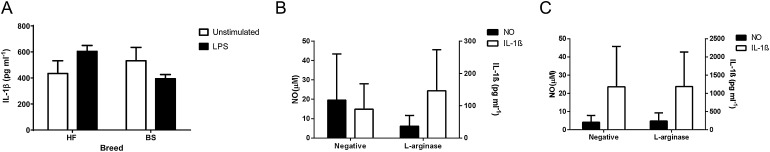
NO blockade partially restores IL-1β Brown Swiss levels to those similar to Holstein Friesian macrophages. MØ derived from PBMC isolated from BS and HF cattle were adhered overnight and stimulated with 2% medium or (A) LPS (5 μg ml^−1^) for 24 h. Supernatants were collected and analysed for bovine IL-1β by ELISA. Absorbance was measured using a SpectraMax M2 plate reader. RNS was inhibited in MØ cultures from (B) BS and (C) HF with l-arginase in the presence of Zymosan (1 mg ml^−1^) for 24 h after adherence of MØ. Bovine IL-1β levels were determined by ELISA and RNS by addition of equal volumes of Griess reagent A and B and compared to 2-fold Nitrite standard curve. Absorbance was measured for both assays using a SpectraMax M2 plate reader. All data were post analysed with GraphPad Prism using two-way non-repeated measures ANOVA testing with Bonferroni post-*hoc* correction.

Having established that MØ and milk-derived cells from BS cows seem to produce more ROS/RNS compared to cells derived from HF cows, we next wanted to establish whether this increased reactivity is a general sign for an increased innate immune response in BS derived cells, and extends to other mediators of the innate immune response, such as production of IL-1β. Interestingly, stimulation of MØ derived from HF, but not BS cows with LPS resulted in increased amounts of IL-1β in the supernatant (Fig. 4A). Recently, a link between NOS and activation of the inflammasome was described (Mao et al., 2013, Mishra et al., 2013), indicating that production of RNS inhibits inflammasome activation. To assess whether such mechanism may also account for the reduced ability of BS MØ to produce IL-1β in response to LPS, we next inhibited RNS production using l-arginase (Garvey et al., 1997), which converts arginine into ornithine and urea, thus removing the substrate of iNOS. Indeed, inhibition of RNS production by addition of l-arginase partially restored the ability of BS MØ to produce IL-1β (Fig. 4B) indicating that activation of the inflammasome is potentially impaired due to high ROS/RNS production. In contrast, no effect was seen when using HF MØ (Fig. 4C), which was potentially due to the low production of RNS in the first instance by these cells.

### 3.5 Autophagy inhibition increases LPS induced IL-1β secretion from both Brown Swiss and Holstein Friesian macrophages

**Fig. 5:**
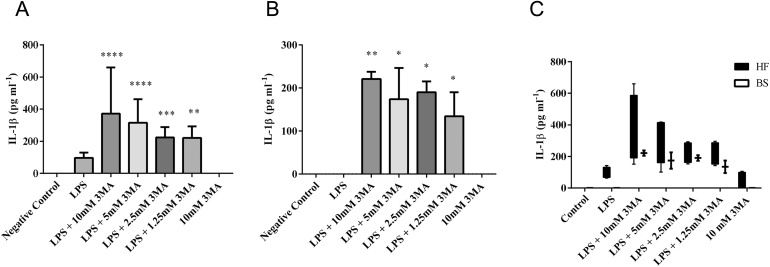
Autophagy inhibition increases LPS induced IL-1β secretion from both Brown Swiss and Holstein Friesian macrophages. 3MA inhibits autophagy by blocking autophagosome formation via the inhibition of type III Phosphatidylinositol 3-kinases (PI-3 K), leading to a down-regulation of inflammaosome activation, and thus IL-1β production. To assess whether MØ of both breeds have the capability to respond with IL-1 production to inflammasome activation, MØ were derived from PBMC isolated from (A) HF and (B) BS and adhered overnight prior to inhibition of autophagy with 3MA (from 10 mM to 1.25 mM) in the presence of LPS (5 μg ml^−1^) for 24 h. Supernatants were collected and analysed for bovine IL-1β by ELISA. Absorbance was measured using a SpectraMax M2 plate reader. All data were post analysed using GraphPad Prism using two-way non-repeated measures ANOVA with Bonferroni post-*hoc* testing.

Inflammasome activation and autophagy are thought to be two mutual exclusive processes within cells (Harris, 2011), and inhibition of autophagy has been described to increase inflammasome activation, resulting in increased IL-1β release (Harris, 2011). To further assess whether BS MØ have a generally impaired IL-1β production, autophagy was blocked in MØ of both breeds before cells were stimulated. As seen before, stimulation of MØ of either breed with LPS alone induced very little increase in IL-1β secretion compared to negative controls. Inhibition of autophagy before LPS stimulation increased IL-1β secretion by MØ from both breeds in a dose-dependent manner (Fig. 5A and B). However, the effect was more pronounced in HF MØ (Fig. 5C).

### 3.6 Phagocytosis and bacterial killing is enhanced in Brown Swiss macrophages compared to Holstein Friesian macrophages

**Fig. 6:**
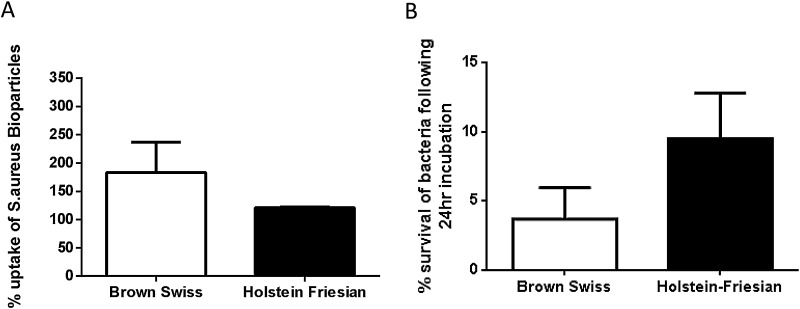
Phagocytosis is enhanced in Brown Swiss macrophages compared to Holstein Friesian macrophages. (A) MØ derived from PBMC isolated from BS and HF cattle were incubated with Alexa-594 labelled *S.a* bioparticles for up to 60 min before fixing with 4% PFA to suspend phagocytosis. Cells were analysed with BD FACS Calibur recording 10,000 events using Cell Quest Pro. Data are presented corrected against MFI at 0 min and post analysed with FlowJo v10 and GraphPad Prism using unpaired students *t*-test with Bonferroni post-*hoc* correction applied. (B) MØ were derived from PBMC isolated from BS and HF and adhered overnight prior to exposure to *S. typhimurium* (10CFU) for 1 h. Supernatants were collected, cells were washed and culture was continued in the presence of Gentamycin for 24 h. Supernatants (1 h and +24 h) and lysed MØ preparations where serially diluted 10-fold and plated for counting. Data were collated and post analysed using GraphPad Prism using unpaired students *t*-test with Bonferroni post-*hoc* testing.

As the increased production of ROS/RNS by BS MØ potentially indicated a greater anti-bacterial response, we finally assessed whether these differences could be attributed to phagocytic activity and bacterial killing. To do so, MØ of both breeds were assessed their ability to phagocytose *S.a.* bio-particles. After 60 min BS MØ engulfed more bio-particles compared to HF MØ, after normalisation to background phagocytosis (Fig. 6A). Lastly, we aimed to investigate whether all the parameters analysed increased the capability of BS MØ to kill bacteria. Therefore, MØ from both breeds were co-cultured with *S. typhimurium* and bacterial killing determined by recovered bacterial growth as described. Significantly fewer bacteria survived in BS MØ compared to HF MØ cows after 24 h of culture post-bacterial incubation (Fig. 6B).

## 4 Discussion

In the present study, we examined potential functional differences to investigate anecdotal evidence suggesting BS cows are more resistant to bacterial infection compared to HF cows. We initially concentrated on the production and release of direct anti-bactericidal effectors indicative of oxidative burst and production of nitrogen species: ROS and RNS. Differences which we observed in blood derived cells were also mirrored in milk derived cells, leading us to investigate other immune parameters; production of pro-inflammatory cytokine and indicator of inflammasome activation, IL-1β, effects of RNS on IL-1β production, the ability of MØ from both breeds to phagocytose and finally the capacity of MØ to exert bactericidal effects on live bacteria.

An intriguing picture has emerged from this work, indicating that there is some functional evidence to support anecdotal differences between BS and HF cattle. Finding that BS MØ or milk derived MØ produce more ROS in response to the same stimulation compared to HF MØ indicates that differences between breeds may be reflected at the genetic level. Cattle breed resistance to infectious disease is not a new concept, there are descriptions detailing specific breed or traits for protection against infection with *Mycobacterium bovis* and *Brucella* spp. (O’Reilly and Daborn, 1995, Paixao et al., 2006, Vordermeier et al., 2012). Indirectly there are reports that BS and HF cattle respond differently to *S. typhimurium* in the production of free radicals; a study by Norimatsu et al. (2004) found HF MØ to be non-responsive however an independent study by Werling et al. (2004) showed BS MØ to produce high levels of RNS. We also observed greater ROS and RNS production from BS MØ with cells derived from age and lactation matched HF cows to help negate location, herd, and husbandry and pregnancy status differences not accounted for by the earlier studies.

We also examined the production of the pro-inflammatory cytokine, IL-1β, as a marker of inflammasome activation. We observed that HF MØ produce higher IL-1β levels compared to those derived from BS cows.

Making use of recent links between RNS, autophagy and IL-1β secretion, blocking both RNS and autophagy modulated IL-1β. Inhibition of autophagy by means of 3MA treatment exerted a dose-dependent increase in IL-1β production in both cattle breeds; however the blockade of RNS synthesis by depletion of l-arginine allowed a recovery or restoration of IL-1β responses in BS MØ only. These data suggest that RNS synthesis and associated negative feedback on IL-1β production may be more efficient in BS cattle. Additionally it appears that autophagy plays a similar role in the inhibition of inflammasome mediated IL-1β cleavage and release in both breeds, based on that MØ derived from BS and HF cows responded equally to 3MA blockade.

On determining these differences in secreted or released molecules upon stimulation of MØ derived from both cattle breeds, we sought to identify if these disparities followed through to bacterial killing. Mo subsets were found to be similar between breeds, yet *S.a.* bio-particle uptake was greater for BS MØ compared to HF MØ. Furthermore, BS MØ were better able to destroy live bacteria as demonstrated by decreased *S. typhimurium* survival rates after exposure to MØ from both breeds.

Our study examines several aspects of innate immune activation involved in bactericidal effects where BS MØ consistently out-performed HF counterparts. We postulate that these differences may be explained at the genetic level and governed by genes which directly affect recognition and uptake of bacteria; PRR such as C-type Lectins or TLRs, those involved in the production of ROS and RNS and finally genes pertaining to the activation of the inflammsome and IL-1β cleavage. Indeed, if such genetic differences would be identified, our assays would provide means to link genotypes with phenotypes by relatively easy to perform and cost-effective assays. Previous studies have suggested that polymorphsims on TLR genes may confer disease susceptibilities or resistance (Jann et al., 2009, Jann et al., 2008), thus future studies might be best placed to investigate both functional and genetic differences. Our finding that BS cattle were consistently lower over the study period for SCC detected in milk and were better able to kill live bacteria warrants further investigation as a trait indicative of increased immune function, irrespective of breed, and would potentially help to explain inflammation responses in different breeds. Indeed, some of these responses may be perpetuated or made more severe via a prolonged, “false” innate immune response, leading to increased tissue damage. Thus, it would perhaps be pertinent to include such immune function traits in combination with known production parameter traits to improve existing dairy and beef herd stocks in their genetic resistance to (bacterial) infection. Most importantly however, our data clearly show that analysis of immune responses may not be translated between breeds of the same species, and this should be kept in mind when assassing responses to pathogens as well as to vaccines.

## Conflict of interest

The authors declare no conflict of interest. All funding bodies have been named in the acknowledgements.
